# Learning from the U.S. Department of Veterans Affairs Quality Enhancement Research Initiative: QUERI Series

**DOI:** 10.1186/1748-5908-4-13

**Published:** 2009-03-06

**Authors:** Ian D Graham, Jacqueline Tetroe

**Affiliations:** 1 Knowledge Translation Portfolio, Canadian Institutes of Health Research, Ottowa, Canada

## Abstract

As the recent collection of papers from the Quality Enhancement Research Initiative (QUERI) Series indicates, knowledge is leading to considerable action in the United States (U.S.) Department of Veterans Affairs (VA). The QUERI Series offers clinical researchers, implementation scientists, health systems, and health research funders from around the globe a unique window into the both the practice and science of implementation or knowledge translation (KT) in the VA. By describing successes and challenges as well as setbacks and disappointments, the QUERI Series is all the more useful. From the vantage point of Canadian KT researchers and officials at a national health research funding agency, we offer a number of observations and lessons that can be learned from QUERI.

"Knowledge, if it does not determine action, is dead to us."

Plotinus (Roman philosopher 205AD-270AD)

## QUERI contributions and lessons learned

When taken as a whole, the collection of QUERI Series articles reveals VA's own experiences implementing and integrating knowledge translation into a health care system on a national scale – something few, if any, health systems have attempted in such a systematic and rigorous fashion. The Series highlights VA's paradigm shift to an action-oriented approach that meaningfully engages clinicians, managers, patients/clients, and researchers in research-driven initiatives to improve quality. This shift to co-production of knowledge [1] or Mode 2 knowledge production [2], or as CIHR refers to it, integrated knowledge translation [3,4], was visionary and required transformative change. The VA is to be commended for taking this bold step over 10 years ago, and persisting when most health research funding agencies have emphasized researcher-curiosity driven research, and while many health care systems have yet to fully embrace the need for evidence-informed quality improvement.

The promise of this approach is that by engaging clinicians and managers in the research process, the application of the resulting knowledge will be greater, swifter and sustained. As some of the papers reveal, the benefits in terms of health gains are only now beginning to be seen, which highlights two points: 1) this approach to quality improvement/KT has merit, and 2) systemic change, especially when it involves bringing about change nationally, takes leadership, time, persistence and much patience.

From an implementation science perspective, of particular note as revealed in the papers by Stetler and colleagues [5], is that the QUERI program worked hard to apply implementation science in designing the implementation of the program. This involved many factors, such as: QUERI serving as the change agent and providing leadership; designing guiding frameworks (e.g., six-step QUERI process, 4-phase pipeline); developing mechanisms to increase opportunities for service providers and researchers to develop meaningful and sustained interactions and collaborations; focusing on changing attitudes and cultures, developing infrastructure, such an electronic health record that can be used for clinical and research purposes; and developing methods and tools to help all the participants reorient to the new way of doing quality improvement and to embrace implementation research in doing so. Much can be learned from this comprehensive and holistic approach to bringing about health system change to nurture and support evidence informed quality improvement and implementation research.

A major contribution to the field is that the QUERI model divides up the implementation process into manageable and logical steps. This model can be classified as a planned action model, as it is intended to be used to engineer change [6]. This is a very practical framework that should be transferable to other settings and contexts. The only potential deficit of the current version of the framework is that it lacks explicit reference to a step about maintenance or sustainability of quality improvement programs. No doubt, this only reflects the evolutionary nature of QUERI, and that few of its improvement programs have become fully implemented and evaluated. Thus, sustainability issues are only now beginning to emerge. Indeed, the paper by Bowman and colleagues [7] reveals QUERI is thinking about measuring persistence of improvement programs. The 4-phase pipeline framework provides a pragmatic conceptualization of how improvement projects should be implemented. Our only quibble is that the pipeline analogy might be taken to imply unidirectionality and a closed system, even though the QUERI approach is iterative and dynamic, and anything but unidirectional in nature.

Not withstanding potential concerns about the extent of the transferability of the overall QUERI approach to other health systems and jurisdictions, QUERI makes important and useful contributions to the global implementation toolkit. These include: the QUERI framework [8], methods (e.g. methods for conducting a formative evaluation [9]), process for developing implementation interventions [10], methods for measuring persistence [7], approaches for considering economic considerations [11], process for developing a national dissemination or spread plan [12], templates (e.g. QUERI Service-Directed Project template that provides guidelines for how to write a protocol for an implementation study) [8], QUERI implementation study checklist for reviewers [8], and tools (e.g., glossary) [8].

These papers provide rich detail, which allow implementers and implementation researchers to make use of these frameworks, methods, and tools. Other papers in the QUERI Series describe implementation intervention projects and their effectiveness. These papers are contributing to the implementation intervention knowledge base. Table 1 illustrates the mix of papers addressing cross-cutting issues [5,7-13] while Table 2 lists the empirical papers [7,9,10,12,14-17] and how each fits into the QUERI step/phase framework.

**Table 1:** QUERI Series articles addressing cross-cutting conceptual or methodological issues

Overview of QUERI approach and framework [8]
Ethical and Institution Review Board issues around implementation research [13]

Process for developing an implementation intervention [10]

A framework for how economic considerations should be integrated into the field of implementation [11]

Measuring persistence – what to measure, when, how and how to get it funded [7]

How to conduct a formative evaluation [9]

Overview of approach to implementing QUERI [5]

How to develop a national dissemination plan [12]

**Table 2:** QUERI Series articles reporting empirical studies

	Phase 1 Single site pilot	Phase 2 Small-scale, multi-site implementation trial	Phase 3 Large-scale, multi-region implementation trial	Phase 4 System wide roll-out
Step 1 Select disease/conditions/patient populations	Goetz et al. Implementing and evaluating a regional strategy to improve testing rates in VA patients for HIV [15] Krein et al. Improving eye care for veterans with diabetes [14] Smith et al. Developing a national dissemination plan for collaborative care for depression [12]			

Step 2 Identify evidence based guidelines	Goetz et al. Implementing and evaluating a regional strategy to improve testing rates in VA patients for HIV [15] Krein et al. Improving eye care for veterans with diabetes [14] Smith et al. Developing a national dissemination plan for collaborative care for depression [12]			

Step 3 Measure and diagnose quality/performance gaps	Goetz et al. Implementing and evaluating a regional strategy to improve testing rates in VA patients for HIV [15] Krein et al. Improving eye care for veterans with diabetes [14] Smith et al. Developing a national dissemination plan for collaborative care for depression [12]			

Step 4 Implement improvement program	Brown et al. EQUIP: Implementing chronic care principles for improved care for schizophrenia [16] Goetz et al. Implementing and evaluating a regional strategy to improve testing rates in VA patients for HIV [15] Krein et al. Improving eye care for veterans with diabetes [14] Smith et al. Developing a national dissemination plan for collaborative care for depression [12]	Brown et al. EQUIP: Implementing chronic care principles for improved care for schizophrenia [16] Wallace and Legro. Project to increase vaccination rates in high risk veterans [9] Sales et al. Implementing electronic clinical reminders for lipid management for patients with ischemic heart disease [17] *Curran et al. A process for developing an implementation intervention [10] *Bowman et al. Measuring persistence of implementation [7]		Smith et al. Developing a national dissemination plan for collaborative care for depression [12]

Step 5 Assess improvement program feasibility, implementation, impacts on patients, family, system outcomes	Krein et al. Improving eye care for veterans with diabetes [14]	Brown et al. EQUIP: Implementing chronic care principles for improved care for schizophrenia[16] Wallace and Legro. Project to increase vaccination rates in high-risk veterans [9] Sales et al. Implementing electronic clinical reminders for lipid management for patients with ischemic heart disease [17]		Smith et al. Developing a national dissemination plan for collaborative care for depression [12]

Step 6 Assess improvement program impact on HRQoL				Smith et al. Developing a national dissemination plan for collaborative care for depression [12]

* indicates data from this step/phase referred to in a thematic paper

This thumbnail sketch of the QUERI Series reveals that the concentration of effort has focused on Steps 1–5 of the QUERI process model and Stages 1–2 of the QUERI pipeline, although work related to Stages 3 and 4 is underway as alluded to in many papers. In the coming years, considerable attention will be focused on QUERI research, assessing impacts on patients and system outcomes (step 5) and evaluating impacts on health-related quality of life (step 6). This will provide the ultimate validation of QUERI's effectiveness. While some studies of multi-region and national roll-out of improvement projects (Phases 3 and 4) are being launched or are underway, their results will be keenly anticipated. This will provide direction for how to facilitate widespread dissemination and uptake of best practices on a larger scale.

When the Series is taken as a whole, QUERI can be considered a national case study that reveals that changing a health care and research system by reorienting efforts toward the implementation of best practices is a complex, long, and never-ending process, but the potential gains in health outcomes makes it worth it. The QUERI Series also illustrates the value of working top-down and bottom-up, if not simultaneously, then iteratively. In other words, clear vision and leadership, supported by frameworks and tools, as well as a responsive collaborative team-focused work force is a winning combination for innovation within the health system.

## Importance of collaboration and sharing knowledge

Implementation is a social activity that is dynamic and interactive, and hence the value of conducting assessments of barriers and formative evaluations is that they provide critical information when designing, adjusting, and evaluating implementation interventions. The QUERI researchers are making important contributions to these methods.

For implementation researchers adopting a collaborative or participatory approach, developing and maintaining the right partnerships with clinical leaders and service providers from the beginning is critical for success. Potential knowledge-users such as these have knowledge and experience that can "grease" the implementation wheels and provide a road map to the potential mine fields inherent in attempting to introduce change in any organization. Furthermore, opportunities for this sort of collaboration can be increased through programs or mechanisms that provide incentives to partner. For those implementation researchers not already embedded in a clinical setting, as VA researchers are, the introduction of adequately resourced requirements for true partnerships with clinical leaders and service providers can provide the incentive for truly collaborative research, or integrated KT.

As Chaney and colleagues [13] pointed out, there are some unique ethical issues related to implementation research that deserve greater attention and clarity. One of these issues that is particularly interesting was alluded to in several papers [5,9,10,13,14] – the dual role of researcher and implementation facilitator, which can be challenging and confusing for the researcher and the study setting by blurring the line between research and traditional quality improvement initiatives. QUERI researchers need to describe their experiences with managing this dual role so that we can all learn from them. With all of these ethics issues, implementation researchers need to be working with ethics boards to improve understanding of evidence-based quality improvement research and differences between it and traditional health services research.

## Take-home messages

One take-home message for health systems and health research funders is that implementation (the doing of quality improvement/KT) and implementation science (the study of it) must be recognized as long-term investments, and resources must be devoted to them specifically. Perhaps unique because the VA is an integrated health and research system, it is still worth noting that health systems should assume responsibility for implementation of best practice, but should also support (with in-kind contributions and funding) research on how best to implement research, as health systems and patients are the ultimate beneficiaries of effective care. Health systems must view and fund clinical and health services research, research on the science of implementation, and implementation as core infrastructure.

For health research funders, the messages are clear. They should create incentives to encourage collaborative and action-oriented research (mode 2 knowledge production) that meaningfully engages clinicians, patients, and managers of the health system, as well as researchers in finding solutions for the problems those in the health system identify and prioritize. And this responsive research should be adequately funded. There also is a need for more funding for fundamental research on the science of implementation, such as research on the determinants of knowledge use, the effectiveness of interventions to increase the application of knowledge in real-world settings, and studies of the sustainability of improvement efforts. The QUERI Series also reveals that funding agencies should consider that Steps 1–3 can be undertaken as legitimate research endeavors (e.g., selecting the disease, conditions, patient populations, thus conducting a needs assessment); identifying evidence-based guidelines (e.g., locating and assessing the evidence to be implemented, adapting the evidence as necessary to the context); and measuring and diagnosing quality/performance gaps.

While this research is clearly more applied in nature, the findings have the potential for generalizability beyond the local context, and therefore should be eligible for research funding. The VA concept of QUERI Centers focused on a specific patient population or condition and mandated with addressing the designated condition [8] is one that funders also could consider. These centers have a structured program of implementation research that both increases the adoption of best practices, while simultaneously advancing implementation science – a win-win-win situation for funders, researchers and the health services. Research funders and researchers also can learn from QUERI's templates, which provide guidelines for researchers on writing implementation study protocols and guidelines to help reviewers evaluate these protocols [8].

## Conclusion

The QUERI Series is an interesting mix of methods papers and implementation studies, and provides insight into a systems approach to implementation. The methods papers have much to offer in terms of how to conceptualize and undertake implementation and implementation research at the macro (implementing the QUERI at a national or system level), meso (organizational change), and the micro (influencing provider and patient/consumer behavior) level. While all the data are not yet in on the overall effectiveness and impact of the QUERI program, there is much to learn from this living implementation laboratory. We are fortunate that QUERI researchers have documented and shared their processes, methods and experiences. After reviewing the papers in the Series, health systems and health research funders in all jurisdictions should carefully consider what aspects of QUERI they could and should emulate or adapt.

## Competing interests

The authors declare that they have no competing interests.

## Authors' contributions

IDG and JT contributed to all phases of manuscript development. Both authors read and approved the final manuscript.
